# An improved technical trick for identification of the thoracodorsal nerve during axillary clearance surgery: a cadaveric dissection study

**DOI:** 10.1186/s13037-018-0164-2

**Published:** 2018-06-26

**Authors:** Dimonge Joseph Anthony, Basnayaka Mudiyanselage Oshan Deshanjana Basnayake, Nambunanayakkara Mahapalliyaguruge Gagana Ganga, Yasith Mathangasinghe, Ajith Peiris Malalasekera

**Affiliations:** Department of Anatomy, Faculty of Medicine, University of Colombo, Kynsey Road, Colombo, 00800 Sri Lanka

**Keywords:** Thoracodorsal pedicle, Lateral thoracic vein, Anatomical landmark, Axillary clearance

## Abstract

**Background:**

Accurate anatomical landmarks to locate the thoracodorsal nerve are important in axillary clearance surgery.

**Methods:**

Twenty axillary dissections were carried out on ten preserved Sri Lankan cadavers. Cadavers were positioned dorsal decubitus with upper limbs abducted to 90^0^. An incision was made in the upper part of the anterior axillary line. The lateral thoracic vein was identified and traced bi-directionally. The anatomical location of the thoracodorsal nerve was studied in relation to the lateral border of pectoralis minor and from a point along the lateral thoracic vein, 2 cm inferior to its confluence with the axillary vein.

**Results:**

The lateral thoracic vein was invariably present in all the specimens. All the lateral thoracic veins passed lateral to the lateral border of pectoralis minor except in one specimen, where the lateral thoracic vein passed along its lateral border. The thoracodorsal nerve was consistently present posterolateral to the lateral thoracic vein. The mean distance to the lateral thoracic vein from the lateral border of pectoralis minor was 28.7 ± 12.6 mm. The mean horizontal distance, depth, and displacement, from a point along the lateral thoracic vein, 2 cm inferior to its confluence with the axillary vein to the thoracodorsal nerve were 14.5 ± 8.9 mm, 19.7 ± 7.3 mm and 25 ± 5 mm respectively. The thoracodorsal nerve was found in a posterolateral direction, at a 54^0^ ± 12^0^ angle to the horizontal plane, 95% of the time.

**Conclusions:**

The lateral thoracic vein is an accurate guide to the thoracodorsal nerve. We recommend exploring for the thoracodorsal nerve from a point 2 cm from the confluence of the lateral thoracic vein and the axillary vein for a distance of 25 ± 5 mm in a posterolateral direction, at a 54^0^ ± 12^0^ angle to the horizontal plane.

## Background

The thoracodorsal nerve (TDN) is a branch of the posterior cord of the brachial plexus. The bulk of the nerve fibers arise from the C7 nerve root [1]. It emerges between the upper and lower subscapular nerves. The TDN passes behind the axillary vein. It first appears in the axilla deep to the lateral thoracic vein (LTV), which is a consistent tributary of the axillary vein [2]. Initially it accompanies the subscapular artery. Then it closely follows the thoracodorsal artery, which is a branch of the subscapular artery. Less commonly the thoracodorsal artery directly arises from the lateral thoracic artery [3, 4]. The thoracodorsal nerve, thoracodorsal artery and thoracodorsal vein are collectively known as the thoracodorsal pedicle (TDP). The TDP descends on the posterior wall of the axilla. The TDN rarely runs 2 to 3 cm medial to the thoracodorsal artery and vein [5]. This phenomenon is sometimes associated with double axillary veins [5]. The TDN ends by supplying the latissimus dorsi muscle from its deep surface. The latissimus dorsi is invariably supplied by the TDN [4]. The TDN may also supply the teres major in 10–20% of the subjects [6].

The first reported latissimus dorsi musculocutaneous flap was used in chest wall reconstruction following mastectomy in 1912 [7]. Over the last few decades, the latissimus dorsi flap has gained interest in reconstructive surgeries involving head and neck, chest, abdomen and limbs. Currently the TDN grafts are used in various neurotrauma surgeries for functional restoration. Moreover, the TDN is at risk during oncosurgical axillary clearance procedures. Hence a sound knowledge on the accurate anatomical landmarks to locate the neurovascular pedicle is important.

The angular vein, a tributary of the thoracodorsal vein was described in 1993 as a constant anatomical landmark related to the TDN [8]. Due to conflicting evidence in subsequent studies [9, 10], the angular vein did not gain much attention as a reliable guide to the TDP. There is only one published article which describes the LTV as a guide to the TDN [2]. However the exact anatomical relationship between these two structures and the distances have not been studied.

Despite the growing need, safe anatomical landmarks to trace the TDN are still poorly studied in the medical literature. This study is designed to develop a standard protocol to identify the TDN intraoperatively during an axillary dissection based on accurate measurements using the LTV as the reference landmark.

## Methods

A descriptive anatomical study was carried out on self-donated cadavers. The study was ethically approved by the Ethics Review Committee, Faculty of Medicine, Colombo and was conducted in accordance with the guidelines set forth by the Declaration of Helsinki.

Twenty axillary dissections were carried out on ten preserved Sri Lankan cadavers. Both male and female cadavers of Sri Lankan nationality were selected randomly. Cadavers with deformed axillary regions or scars in the axillae were excluded from the study. All selected cadavers were preserved with conventional arterial injection method using 10% formalin as the main preservative. Cadavers were positioned dorsal decubitus, with arm abducted to 90^0^.

**Fig. 1:**
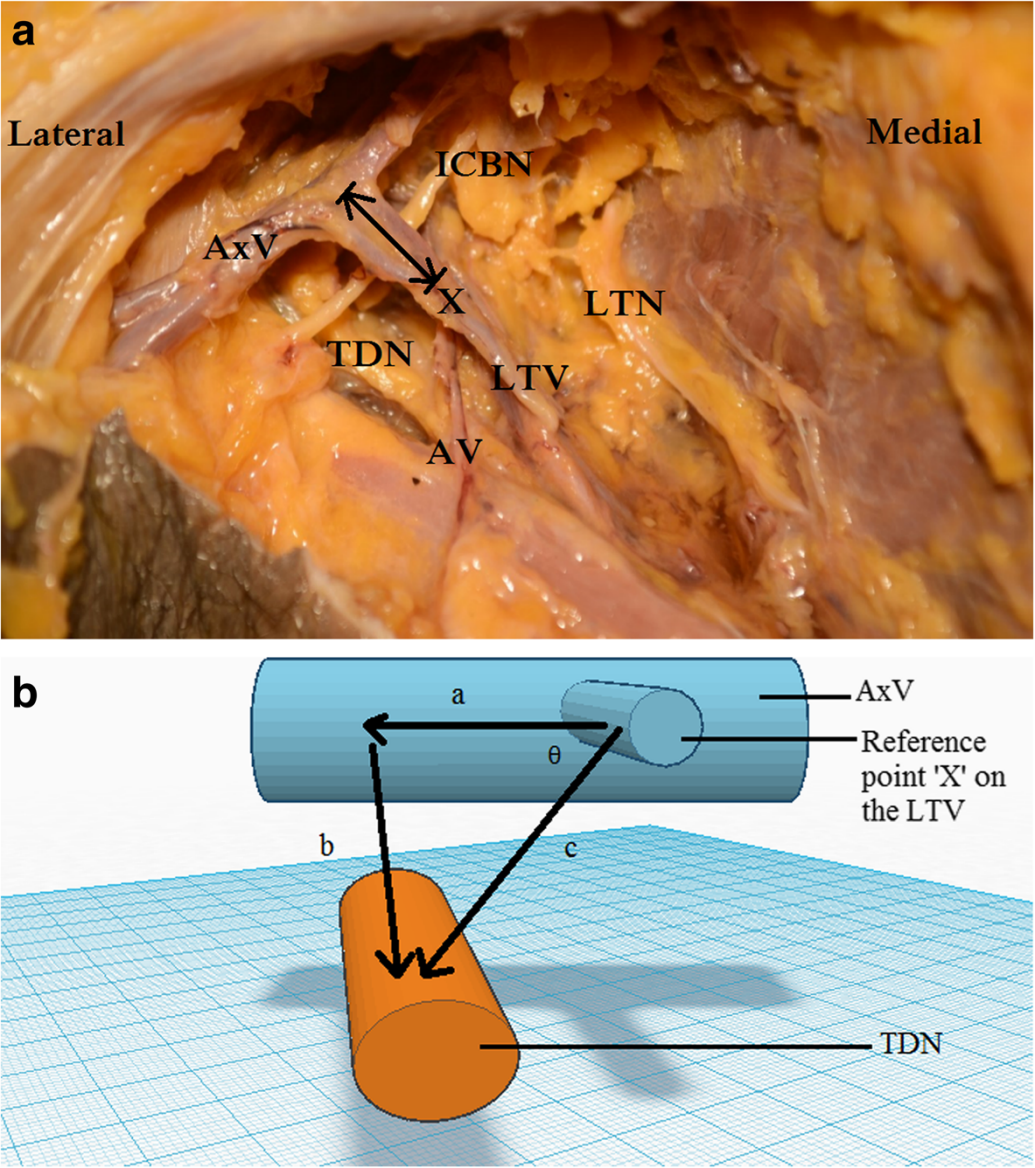
The relationship of the thoracodorsal nerve and the lateral thoracic vein. The image on the left shows a dissection of a right axilla. The image on the right shows a 3D diagram (AV – Angular vein, AxV – Axillary vein, ICBN – Intercostobrachial nerve, LTN – Long thoracic nerve, LTV – Lateral thoracic vein, TDN – Thoracodorsal nerve, X – Reference point, a - horizontal distance, b – depth, c – displacement, θ – angle with the horizontal plane)

An incision was made along the upper part of the anterior axillary line. Pectoralis major was retracted upwards and medially. Pectoralis minor was identified and the axillary fascia divided. The axillary vein, and the termination of the LTV on to it was identified. A blunt dissection was carried out along the axillary vein to identify its tributaries and the accessory LTVs. The TDP was identified and preserved. The connective tissue surrounding the veins and pedicles were minimally disturbed so as to preserve the anatomical relationships. The anatomical location of the TDN was studied in relation to the lateral border of pectoralis minor and from a point along the LTV, 2 cm inferior to its confluence with the axillary vein (reference point x). Following measurements were recorded using a Vernier caliper [Manufacturer- Mitutoyo (Kanagawa- Japan) [Model No- 505-633-50] (Fig. 1).

1. The distance (d) from the lateral border of the pectoralis minor to the reference point X (a point along the LTV, 2 cm inferior to its confluence with the axillary vein).
2. The horizontal distance (a) to TDN from the reference point X.
3. The depth (b) to the TDN from the reference point X.

The neurovascular structures were kept under slight tension to avoid kinking. In relation to d above, if the LTV was medial to the lateral border of the pectoralis minor, the value was denoted with a minus, and vice versa. The displacement and the angle to the horizontal plane between TDN and LTV, from reference point x were calculated. Data were analyzed using Statistical Package for Social Sciences (IBM SPSS) version 21 with a priori alpha of.05.

## Results

All the cadavers had bilateral single axillary veins. The LTV was present in all the specimens. All the LTVs passed lateral to the lateral border of pectoralis minor except in one specimen, where the LTV passed along the lateral border of pectoralis minor. The TDN was consistently present posterolateral to the LTV. The accessory LTVs were found in bilateral axillae of two dissected cadavers (*n* = 2, 20%). The accessory LTVs were much smaller in diameter when compared to the principal LTVs so as to not cause any confusion in recognizing the main LTV. The accessory veins were seen proximal to the junction of the main trunk of the LTV with the axillary vein in all specimens. They had no communications with each other and drained independently to the axillary vein. Three of the accessory LTVs were medial to the lateral border of pectoralis minor and one was lateral. The mean distance between the proximal accessory LTVs and the lateral border of pectoralis minor was 15 mm (±4 mm). The distal one was situated 47 mm distal to the lateral border of pectoralis minor.

The most commonly observed orientation of neurovascular structures during the study is illustrated in Fig. 1. The measured distances (d, a, b) are given in Table 1. The distances (d, a, b) were examined to determine the extent to which the assumption of normality was met. The Table 1 also shows skewness, kurtosis and the Shapiro-Wilk test of normality. The results suggest that normality is a reasonable assumption. Independent sample *t*-tests were conducted to compare the distances (d,a,b) on left and right sides. There were no statistically significant differences in any of the above mentioned distances on left and right axillae (*p* > .05). The mean distance to the LTV from the lateral border of pectoralis minor was 28.7 ± 12.6 mm. The mean horizontal distance, depth, and displacement, from reference point X to the TDN were 14.5 ± 8.9 mm, 19.7 ± 7.3 mm and 25 ± 5 mm respectively. The TDN was found 54^0^ ± 12^0^ to the horizontal plane, in a posterolateral direction, 95% of the time. The displacement and angle were calculated utilizing the measurements a and b and Pythagoras theorem.

**Table 1:** The summary statistics of the horizontal distance (a) and the depth (b) from reference point X to the TDN and the distance to the LTV from the lateral border of pectoralis minor (d) (df - degree of freedom, LTV - lateral thoracic vein, reference point X - a point along the LTV, 2 cm inferior to its confluence with the axillary vein, SD – standard deviation, SE - standard error, TDN - thoracodorsal nerve)

Measured distances	Mean (mm)	SD (mm)	95% confidence interval (mm)	Range (mm)	Skewness	Kurtosis	Shapiro-Wilk test (df = 20)
Distance to the LTV from the lateral border of pectoralis minor (d)	28.7	12.6	22.8–34.6	0–47	−.543 (SE = .512)	−.081 (SE = .992)	.956 (*p* = .465)
Horizontal distance from reference point X to the TDN (a)	14.5	8.9	10.3–18.6	2–31	.559 (SE = .512)	−.718 (SE = .992)	.926 (*p* = .132)
Depth from reference point X to the TDN (b)	19.7	7.3	16.3–23.1	8–34	−.071 (SE = .512)	−.620 (SE = .992)	.964 (*p* = .624)

## Discussion

No variations of the axillary vein contradicting the textbook description were found in the dissected specimens [11]. An English study conducted in 100 patients with breast carcinoma undergoing staging axillary dissections found that 10% (*n* = 10) of the subjects were having double axillary veins [5]. Duplications of the axillary arteries were absent in the present study. The accessory LTVs were described by a similar study conducted on 100 patients undergoing axillary lymph node dissections [12]. They also had encountered independent veins draining to axillary vein. But the reported incidence was much lower (1%, *n* = 1) compared to the present study (20%, *n* = 2). In accordance with the current study, TDN was consistently present posterolateral to the confluence of the LTV with the axillary vein [12]. In all cases, the TDN was accompanied by corresponding vessels closely. There were no stray nerves as described by the aforementioned study where 5% (*n* = 5) patients had the TDN running 2 to 3 cm medial to the thoracodorsal vessels [5]. In these patients, the TDN converged to the corresponding vascular pedicle only adjacent to the point of insertion to the muscle. A similar Malaysian study concluded that in 14.3% (*n* = 5) of the cases, the TDN did not accompany the thoracodorsal vessels throughout [13]. However these stray nerves in the former study were associated with double axillary veins in 2 patients. Absence of such variations of the axillary vein in the present study population might be the explanation for homogeneous relationship of structures within the TDN. The recommended measurements of our study therefore should be applied in patients with single axillary veins. The LTV maintained its anteromedial relationship to the TDN in 100% (*n* = 20) cadavers in the current study. Conversely the TDN and the LTV was found to be in the same plane in a minority (36.4%, *n* = 36) in a Brazilian surgical anatomical study [12].

The LTV has been recently described as a landmark to identify the TDN. In 2012 a group of Malaysian surgeons stated:

“The axillary vein is then dissected downwards and laterally along its course towards the arm. The lateral thoracic vein appears at the antero-inferior aspect of the axillary vein about 2 to 3 cm lateral to the chest wall. This is the landmark vein for the TDN. The TDN constantly lies just underneath the lateral thoracic vein. Careful dissection and ligation of the lateral thoracic vein exposes the TDN simply and effortlessly [2]”

In keeping with the above study, the present study findings also confirm that the LTV is superficial to the TDN reinforcing its suitability as a landmark structure. In 2001, Khatri et al. stated that the LTV is closely related to the TDN [14]. According to their experience the TDN was situated approximately 1 cm lateral and somewhat deep to the LTV. Our study scientifically confirmed that the mean horizontal distance, depth, and displacement, from reference point X to the TDN were 14.5 ± 8.9 mm, 19.7 ± 7.3 mm and 25 ± 5 mm respectively. But they suggested using the anterolateral border of latissimus dorsi to identify the TDN rather than dissecting next to the LTV [14]. In contrast utilizing our method involves less extensive dissection and quicker and safer identification of the TDN.

In 1993 O’Rourke and Layt described the angular vein of axilla (AV) for the first time [8]. Following 11 fresh cadaveric dissections and 30 live axillary dissections, the authors identified this unnamed tributary of the subscapular vein as a consistent relationship to the TDN. The vein was found in 95% (*n* = 41) of the dissections. According to the authors, the AV drained to the subscapular vein to form the thoracodorsal vein, and just distal to the confluence of the veins, the TDN angled over the AV superficially. In 2003, Chan and Tan described the spatial relations of the AV as an important landmark in axillary nodal dissection [9]. But the results of the three studies were conflicting. Still there is confusion over using this vein as an anatomical landmark in axillary dissection [15]. The majority of professionals seem to be reluctant to use this landmark routinely [15].

Hence the LTV is a promising landmark to identify the TDN. Relations of other important structures in the axilla (eg: intercostobrachial nerve, long thoracic nerve) was out of scope of this study. The present study was based on embalmed cadavers. Thus, subtle distortions of anatomy due to postmortem changes cannot be ruled out. Since the sample size was small, population inferences may not be accurate.

## Conclusions

Identifying and preserving the TDN safely is critical in axillary surgery and reconstructive surgeries involving the latissimus dorsi muscle. The LTV is a fairly accurate guide to the TDN. We recommend exploring for the TDN from a point 2 cm from the confluence of the LTV and the axillary vein for 25 ± 5 mm in a posterolateral direction, at a 54^0^ ± 12^0^ angle to the horizontal plane, since 95% of the time the TDN was found within this region. Further studies are necessary to appreciate variations among populations. We recommend further studies to clinically correlate the findings of the present study with routine axillary dissections on living subjects and 3D reconstructive Computed Tomography angiograms.

## Abbreviations

AxV: Axillary vein
ICBN: Intercostobrachial nerve
LTV: Lateral thoracic vein
TDN: Thoracodorsal nerve
TDP: Thoracodorsal pedicle
